# Prevalence of and factors associated with early initiation of breastfeeding among women with children aged < 24 months in Kilimanjaro region, northern Tanzania: a community-based cross-sectional study

**DOI:** 10.1186/s13006-020-00322-8

**Published:** 2020-09-10

**Authors:** Farida Ali, Melina Mgongo, Redempta Mamseri, Johnston M. George, Innocent B. Mboya, Sia E. Msuya

**Affiliations:** 1 Institute of Public Health, Department of Epidemiology and Biostatistics, Kilimanjaro Christian Medical University College (KCMUCo), P. O. Box 2240, Moshi, Tanzania; 2 Better Health for African Mother and Child (BHAMC), P.O. Box 8418, Moshi, Tanzania; 3 Institute of Public Health, Department, Department of Community Medicine, Kilimanjaro Christian Medical University College (KCMUCo), P. O. Box 2240, Moshi, Tanzania; 4 School of Mathematics, Statistics & Computer Science, University of KwaZulu Natal, Private Bag X01, Scottsville, Pietermaritzburg, 3209 South Africa; 5 Department of Community Medicine, Kilimanjaro Christian Medical Center (KCMC), P. O. Box 3010, Moshi, Tanzania

**Keywords:** Breastfeeding, Early initiation of breastfeeding, Prevalence

## Abstract

**Background:**

Early initiation of breastfeeding offers nutritional and immunological benefits to the newborn, which is critical for health and survival. Understanding factors associated with timely initiation of breastfeeding is crucial for healthcare providers and policy-makers. This study aimed to assess the prevalence and factors associated with early initiation of breastfeeding among mothers with children < 24 months of age in the Kilimanjaro region, Northern Tanzania.

**Methods:**

This study utilized secondary data from a cross-sectional survey conducted in April 2016 and April 2017 in the Kilimanjaro region. A multistage sampling technique was used to select study participants and interviewed using a questionnaire. A total of 1644 women with children aged < 24 months were analyzed. Modified Poisson regression models were used to determine factors independently associated with early initiation of breastfeeding, within first hour of life.

**Results:**

The prevalence of early initiation of breastfeeding in the Kilimanjaro region was 70%, ranging from 64% in Same to 80% in Siha districts. The prevalence of early initiation of breastfeeding was lower among women who initiated prelacteal feeding compared to their counterparts (prevalence ratio [PR] 0.42; 95% Confidence Interval [CI] 0.34, 0.53). Likewise, women living in Same and Hai district had lower prevalence of early initiation of breastfeeding compared to women in Rombo (PR 0.8; 95% CI 0.76, 0.93) and (PR 0.89, 95% CI 0.80, 0.98) respectively. Higher prevalence of early initiation of breastfeeding was found in women with primary education compared to those with secondary education (PR 1.09; 95% CI 1.003, 1.18), and among women with two children compared to one child (PR 1.14, 95% CI 1.03, 1.26).

**Conclusions:**

Early initiation of breastfeeding practice was suboptimal in this study. To improve early initiation of breastfeeding, healthcare providers at reproductive and child health clinics and labour wards should discourage women from prelacteal feeding, give more support to women with one child and those with secondary level of education and above. Furthermore, a qualitative study is crucial to understand the reasons for low prevalence of early initiation of breastfeeding in Same and Hai districts.

## Background

Early initiation of breastfeeding refers to initiation of breastfeeding within 1 h after birth [1]. The practice gives the best start in life a chance of infant to receive colostrum as a first diet and confers benefits that last for a lifetime [2]. Colostrum is highly rich in nutrients that help child growth and has antibodies that act as a first vaccine hence protects the newborn from infection [2]. Other benefits for the child include; body temperature and glucose regulation and stimulates more milk production. Early initiation of breastfeeding also introduces skin to skin contact with the mother, which is reported to extend the duration of breastfeeding and contributing to an increase in exclusive breastfeeding. For mothers, early initiation of breastfeeding stimulate contraction of uterus after child birth, which reduces the risk of postpartum hemorrhage [2].

Delayed initiation of breastfeeding increases the risk of neonatal morbidity and mortality. Evidence from a systematic review conducted in Ghana, India, and Tanzania showed that infants who initiated breastfeeding more than 1 h after delivery had 50 and 11% higher risk of cough and breathing difficulties, respectively, during the first 6 months of life compared to those who began within 1 h after birth [3]. Also, neonates who started breastfeeding 2–23 h after birth had a 33% greater risk of dying compared to those who began within 1 h after delivery, and the risk doubles when initiation started after 24 h [4–6].

Despite these benefits of early initiation of breastfeeding, globally, only 42% of the newborns initiated breastfeeding within 1 h after birth in 2018 [2]. In the WHO European region, the overall prevalence of early initiation of breastfeeding was 43% [7]. At the same time, in low- middle-income countries, it ranged from 35% in the Middle East and North Africa to 65% in Eastern Southern Africa regions [2]. Prevalence in African countries varies from 34.7% in Nigeria and 87.2% in Sudan [8–13].

Early initiation of breastfeeding is one of the cost-effective interventions to reduce neonatal mortality and morbidity. However, in Tanzania, the prevalence of early initiation of breastfeeding was 51%, while in northern Tanzania, the prevalence was 73.2% ranging between 28% in Simiyu and 80% in Tanga regions, while in Kilimanjaro region the prevalence was 73.7% [14]. These estimates are still far from the set target of 90% by 2020. Tanzania Demographic and Health Survey (TDHS) estimated the neonatal mortality of 25 deaths per 1000 live births, which is higher than the national target of reducing this rate to 16 deaths per 1000 live births by 2020 [14] and Sustainable Development Goals (SDGs) target of 12 deaths per 1000 live births reduction by 2030 [15]. The mortality situation indicates a missed opportunity to use early initiation of breastfeeding as one of the interventions to improve newborn survival.

Tanzania has established the road map strategic plan activities for improving early initiation of breastfeeding. These activities include capacitating healthcare providers in assisting women in initiating breastfeeding within 1 h after birth, improving antenatal care coverage, and training community healthcare workers at all levels on the importance of early initiation of breastfeeding techniques [16]. Despite these interventions, the country is far from reaching the national target of increasing early initiation of breastfeeding from 51% in 2016 to 90% by 2020 [16]. Likewise, in the Kilimanjaro region, where the proportion of hospital delivery is 91%, and delivery by skilled birth attendants is the highest (96%), early initiation of breastfeeding is still suboptimal [14].

Different factors are reported to be positively associated with early initiation of breastfeeding practice, these include; health facility delivery [8, 11], assistance by a skilled birth attendant [10, 17], multiparous [10, 12], and counseling on early initiation of breastfeeding during antenatal care [18]. Factors such as prelacteal feeding [19], caesarean section delivery [11, 12, 16, 20, 21], having no formal education [22], delivering a male child [9], and being unmarried were negatively associated with early initiation of breastfeeding [13, 23]. There are variations of factors that influence early initiation of breastfeeding from one setting to another within and between countries due to differences in cultural, socio-economic conditions, and health inequalities as well as the quality of healthcare services. Understanding context-specific factors will inform targeted interventions to improve early initiation of breastfeeding. This study aimed to assess prevalence and factors associated with early initiation of breastfeeding among mothers with children aged < 24 months in the Kilimanjaro region, Northern Tanzania. The researchers conducted study to determine sociodemographic, reproductive and health utilization services, child characteristics and breastfeeding pattern factors associated with early initiation if breastfeeding.

## Methods

### Study design and setting

This community-based cross-sectional study utilized data from a study that aimed to assess infant and young child feeding practices among children under 5 years of age in the Kilimanjaro region, Northern Tanzania. The study was conducted in April 2016 and April 2017 in six districts out of seven in the Kilimanjaro region, namely; Moshi Municipal, Rombo, Same, Mwanga, Hai, and Siha. We analyzed data for women with children aged 0–23 months, who had ever breastfed and who reported time of initiation of breastfeeding at the time of the interview.

Kilimanjaro region is among the 30 administrative regions in Tanzania Mainland with an area size of 13,250 km^2^. This region is located in the Northern part of the country and has a population of approximately 1,640,000 people, 845,000 (51.6%) being females. More than three-quarters of the population reside in rural areas [24]. Agriculture and livestock keeping are the main economic activities but are complemented by tourism, manufacturing, and business activities. The region has 18 hospitals, 51 health centers, and 333 dispensaries, which are owned by the Government, faith-based organizations, or private institutions/ individuals. These facilities offer preventive, curative, and rehabilitative services, including maternal and child health services such as antenatal care, postnatal care, family planning, delivery, child growth monitoring, and vaccination services among others. According to Tanzania Demographic and Health Survey (TDHS), a vast majority of deliveries (92%) in the region are conducted by skilled birth attendants, but the proportion of women who attend four antenatal care visits is low (25%) [14].

### Study population, sample size, and sampling

**Fig. 1:**
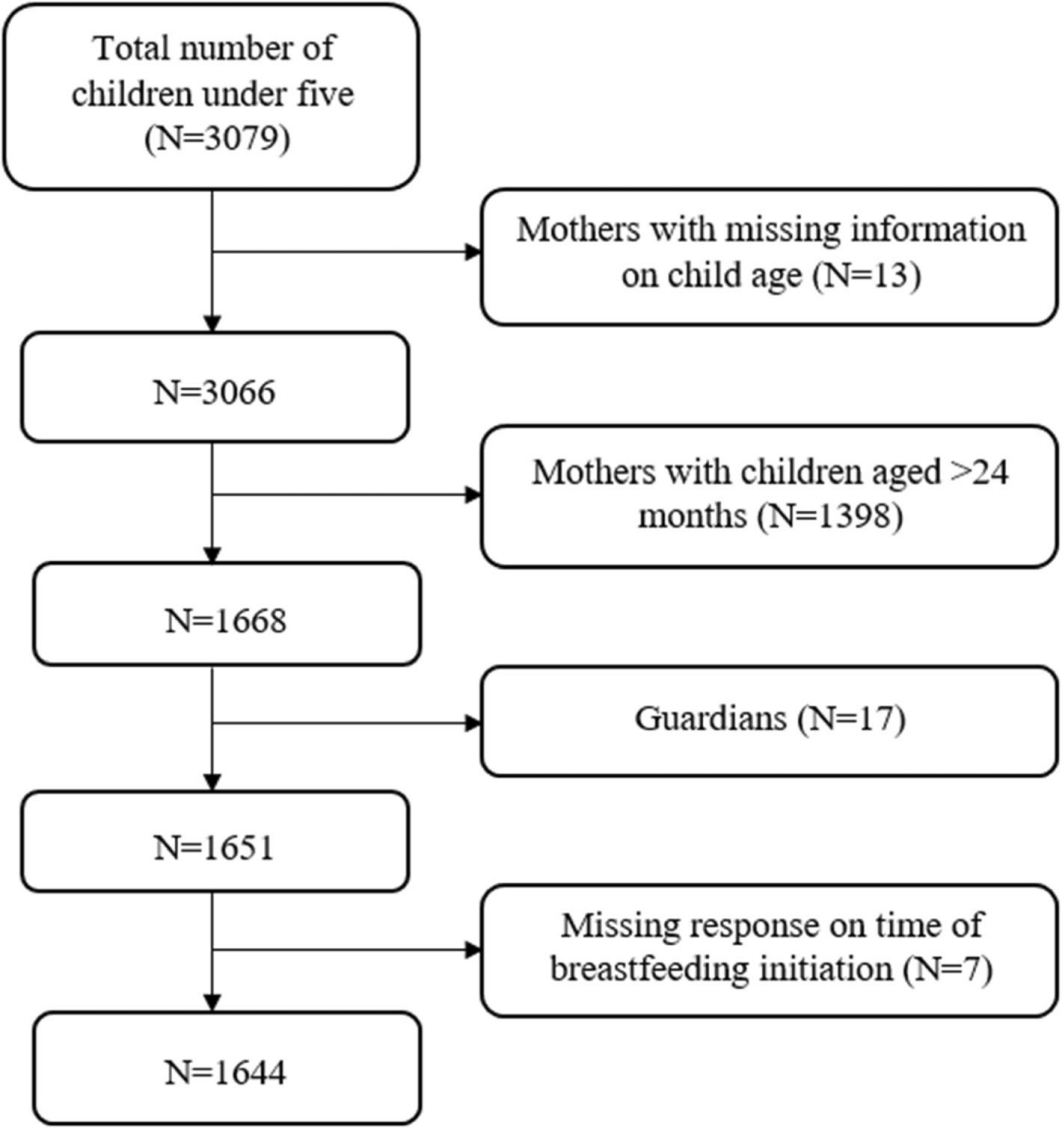
Schematic diagram showing sample size used in the analysis

The parent study enrolled 3079 women with children aged less than 5 years. The current study included women with children aged 0–23 months, with complete information on early initiation of breastfeeding and mothers who came with their infants. Data for 1644 women were, therefore, analyzed in this study (Fig. 1). A multistage sampling technique was used to select participants from each of the six districts. One district (Moshi rural) was purposively excluded because there was an ongoing study investigating anemia in under-five children. In the second stage, three villages (in rural areas) or streets (in urban areas) were randomly selected from each ward. The third stage involved a listing of households with children under 5 years with the help of ward/ village and street leaders/ or link persons, followed by a random selection of households. At the household level, one child was selected. If the household had more than one under-five child, the youngest child was selected.

### Variables

The outcome variable is the early initiation of breastfeeding (EIBF). Early initiation of breastfeeding was measured by asking the mother whether she had ever breastfed the baby, and if yes, time in hours when the child was initiated breastfeeding. This was expressed as a dichotomous variable with 1 representing initiation of breastfeeding within 1 h after birth and 0 if otherwise.

Independent variables analyzed in this study included: sociodemographic characteristics of both the mother and the child, reproductive characteristics, and breastfeeding patterns. Sociodemographic characteristics included the district of residence (Rombo, Moshi Urban, Same, Mwanga, Hai, and Siha); area of residence (urban, rural); maternal age in years (15–24, 25–34, 35–49); marital status (single, married/cohabiting, divorced/widow); education level (no education, primary, secondary and above) and employment status (whether employed or not). Reproductive and Maternal Health Services included parity, number of antenatal care visits (< 4, ≥ 4); breastfeeding counseling during antenatal care visits (yes, no), breastfeeding support after birth (yes, no), place of birth (health facilities, home/others) and level of health facility of delivery (dispensary, health center, hospital). Child characteristics included child age in months (0–11, 12–23), sex (male, female), and birthweight in kilograms (< 2.5 kg and ≥ 2.5 kg) while breastfeeding patterns included prelacteal feeding (yes, no) and use of colostrum (yes, no).

### Data collection method and tools

Face-to-face interviews was the method used to collected data using a questionnaire. The questionnaire collected information on sociodemographic characteristics of the mother and the child, food security at the household level, reproductive health information, breastfeeding patterns, the use of health facilities during pregnancy with the child involved in the study, history of infections, and treatment, customs, and attitude towards early initiation of breastfeeding. The questionnaire was in both English and Swahili languages but administered using the Swahili language, a language spoken by all the local people in this setting. Trained medical students from KCMUCo collected data following participant informed consent.

### Data analysis

Data were cleaned and analyzed using Stata version 15. Continuous variables were summarized using mean/ median with standard deviation/ interquartile range, respectively, and categorical variables using frequencies and percentages. Modified Poisson regression whose measure of association is prevalence ratio (PR) was used as an alternative to classical logistic regression because the prevalence of early initiation of breastfeeding was common (> 10%). Prevalence ratio is defined as the probability of prevalence of diseases among exposures over prevalence of disease among unexposed. Bivariate and multivariable regression models were used to identify factors associated with early initiation of breastfeeding. Independent variables with *p* < 0.05 in the bivariate analysis were entered in the multivariable model to adjust for the potential confounding effect. We used stepwise regression for model building whereby covariates were added and removed from the model by forwarding elimination. The statistically significant *p* - values of Wald test statistics for each covariate determined retention of the variable in the model. We used the likelihood ratio test to compare nested models. The strength of association was expressed using PR and their 95% confidence intervals. Variables with *p* < 0.05 in multivariable analysis were considered to be associated with early initiation of breastfeeding.

## Results

### Respondent characteristics

A total of 1644 mother–child-pairs were included in this analysis. The mean age ± SD of mothers was 28 ± 7 years, and 44.7% were aged between 25 and 34 years. More than three quarters were rural residents, 73.9% were married/ cohabiting with their partners, 62.1% had primary education level, and majorities (86.3%) were not employed. About 60 % of their partners had primary education level (Table 1).

**Table 1:** Sociodemographic characteristics of study participants, *N* = 1644

Variable	Frequency	Percentage
**District of residence**		
Rombo	372	22.6
Moshi urban	170	10.3
Same	355	21.6
Mwanga	179	10.9
Hai	296	18.0
Siha	272	16.6
**Area of residence**^**a**^		
Urban	365	22.2
Rural	1278	77.8
**Maternal age in years**^**a**^		
15–24	609	37.2
25–34	733	44.7
35–49	297	18.1
Mean ± SD	28 ± 7	
**Marital status**^**a**^		
Married	1213	73.9
Single	357	21.7
Divorced/widow	72	4.4
**Maternal education level**^**a**^		
Non-formal	56	3.4
Primary	1020	62.1
Secondary and above	566	34.5
**Employed with salary**^**a**^		
No	1418	86.4
Yes	224	13.6
**Partner education level**^**a**^		
Non-formal	40	2.8
Primary	849	58.9
Secondary and above	552	38.3

^a^Frequencies do not tally to the total due to missing values in these variables

### Reproductive characteristics and utilization of maternal health service use

More than one-third of all mothers had one child with a median parity of 2 (IQR 1, 3). The majority (76.9%) had ≥4 antenatal visits, received breastfeeding counseling at antenatal (64.8%), while 28.5% received breastfeeding support immediately after birth, and 87.1% delivered at the health facilities. Out of 1541 who delivered at the health facilities, 62.2% delivered at the hospital (Table 2).

**Table 2:** Reproductive characteristics of study participants, *N* = 1644

Variable	Frequency	Percentage
**Parity**^**a**^		
1	565	34.4
2	428	26.0
≥ 3	650	39.6
Median (IQR)	2 (1,3)	
**Number of antenatal care visits**^**a**^		
< 4	376	23.1
≥ 4	1254	76.9
**Antenatal care breastfeeding counseling**^**a**^		
No	573	35.2
Yes	1054	64.8
**Place of birth**^**a**^		
Health facility	1418	87.1
Home/Others	209	12.9
**Level of health facility of birth (*n*** **= 765)**^**a**^		
Dispensary	190	12.3
Health center	392	25.5
Hospital	959	62.2
**Breastfeeding support immediately after birth by provider**^**a**^		
No	611	71.5
Yes	244	28.5

^a^Frequencies do not tally to the total due to missing values in these variables

### Child characteristics and breastfeeding patterns

The mean age ± SD of children in this study was 11 ± 7 months, and more than half (52.7%) were aged ≤11 months. Half of all children were females. The vast majority (93%) had a birthweight of 2.5Kg, 95.9% were given colostrum, and only 11% were given prelacteal feeds (Table 3).

**Table 3:** Child characteristics and breastfeeding patterns, *N* = 1644

Variable	Frequency	Percent
**Age in months**		
0–11	872	52.7
12–23	783	47.3
Mean ± SD	11 ± 7	
**Sex**		
Male	814	49.5
Female	829	50.5
**Birthweight in kilograms (Kgs)**^**a**^		
< 2.5	113	7.0
≥ 2.5	1498	93.0
**Breastfeeding initiation**		
Early initiation (within 1 h after birth)	1150	70.0
Late initiation (after 1 h)	494	30.0
**Child given prelacteal feed**^**a**^		
No	1467	89.2
Yes	176	10.8
**Child given colostrum**^**a**^		
No	69	4.1
Yes	1574	95.9

^a^Variables with missing information

### Prevalence of early initiation of breastfeeding

**Fig. 2:**
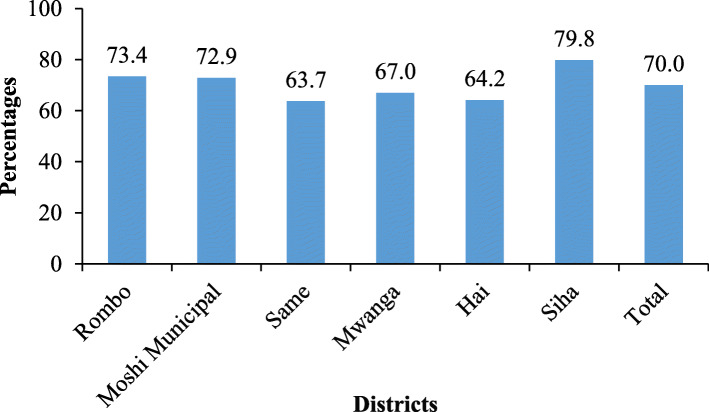
Early Initiation of Breastfeeding by districts in the Kilimanjaro region (*N* = 1644)

The prevalence of early initiation of breastfeeding was 70%. The prevalence ranged between 63.7% in the Same district and 79.8% in the Siha district council (Fig. 2).

### Factors associated with early initiation of breastfeeding

In bivariate analysis, the district of residence, mother’s and partner’s education level, parity, and prelacteal feed were the factors significantly (*p* < 0.05) associated with early initiation of breastfeeding. Prevalence of early initiation of breastfeeding was 13% lower among women in Same and Hai district compared to Rombo district, (PR 0.87; 95% CI 0.79, 0.96) and (PR 0.87, 95% CI 0.79, 0.97) respectively. Prevalence was 12% higher among women who had given birth to two children and 10% higher among women who had given birth more than twice compared to those who gave birth once (PR 1.12; 95% CI 1.02, 1.21) and (PR 1.10; 95% CI 1.01, 1.19), respectively. Women with primary education level had an 11% higher prevalence of early initiation of breastfeeding compared to those who had no formal education (PR 1.11; 95% CI 1.03, 1.19). Likewise, women whose partners had primary education level had an 8% higher prevalence of early initiation of breastfeeding compared to those whose partners had no formal education (PR 1.08; 95% CI 0.89, 1.32). Also, the prevalence of early initiation of breastfeeding was 59% lower among women who introduced prelacteal feeding (PR 0.41; 95% CI 0.33, 0.51) compared to those who did not (Table 4).

**Table 4:** Factors associated with early initiation of breastfeeding, *N* = 1644

Variable	***n***	EIBF ***n*** (%)	CPR (95% CI)	***P***-value	APR (95% CI)	***P***-value
**District**						
Rombo	372	273 (73.4)	1		1	
Moshi urban	170	124 (72.9)	0.99 (0.89, 1.11)	0.914	0.99 (0.89, 1.10)	0.848
Same	355	226 (63.7)	0.87 (0.79, 0.96)	0.005	0.84 (0.76, 0.93)	0.001
Mwanga	179	120 (67.0)	0.91 (0.81, 1.03)	0.138	0.92 (0.81, 1.05)	0.203
Hai	296	190 (64.2)	0.87 (0.79, 0.97)	0.012	0.89 (0.80, 0.98)	0.024
Siha	272	217 (79.8)	1.09 (1.00, 1.19)	0.056	1.07 (0.98, 1.17)	0.156
**Residence**						
Urban	365	255 (69.9)	1			
Rural	1279	895 (70.0)	1.00 (0.93, 1.08)	0.967		
**Maternal age (years)**						
15–24	609	416 (68.3)	1			
25–34	734	513 (69.9)	1.02 (0.95, 1.10)	0.533		
35–49	298	218 (73.2)	1.07 (0.98, 1.17)	0.125		
**Child age (months)**						
0–11	871	607 (69.7)	1			
12–23	773	543 (70.3)	1.01 (0.94, 1.07)	0.819		
**Child sex**						
Female	814	564 (69.3)	1.02 (0.96, 1.09)	0.572		
Male	829	585 (70.6)	1			
**Marital status**						
Married	1213	866 (71.4)	1			
Single	357	239 (67.0)	0.94 (0.86, 1.02)	0.12		
Divorced/widow	72	43 (59.7)	0.83 (0.69, 1.01)	0.07		
**Maternal education**						
No formal	56	40 (71.4)	1.09 (0.92, 1.30)	0.324	1.00 (0.82, 1.24)	0.933
Primary	1020	739 (72.5)	1.11 (1.03, 1.19)	0.005	1.09 (1.003, 1.18)	0.043
Secondary & above	566	370 (65.4)	1		1	
**Employed with salary**						
No	1418	1000 (70.6)	1			
Yes	224	148 (66.1)	0.93 (0.85, 1.03)	0.193		
**Partner education**						
No formal	40	29 (72.5)	1.08 (0.89, 1.32)	0.441	1.04 (0.84, 1.28)	0.743
Primary	849	614 (72.3)	1.08 (1.004, 1.16)	0.038	1.03 (0.60, 1.11)	0.399
Secondary & above	552	370 (67.0)	1		1	
**Parity**						
1	565	364 (64.4)	1		1	
2	428	326 (76.2)	1.18 (1.09, 1.28)	< 0.001	1.12 (1.02, 1.21)	0.011
≥ 3	650	459 (70.6)	1.10 (1.01, 1.19)	0.023	1.03 (0.94, 1.12)	0.523
**Frequency of ANC care**						
< 4	376	255 (67.8)	1			
≥ 4	1254	885 (70.6)	1.04 (0.96, 1.13)	0.319		
**BF counseling at ANC**						
No	573	392 (68.4)	1			
Yes	1054	742 (70.5)	1.03 (0.96, 1.10)	0.388		
**Place of birth**						
Home/others	209	137 (65.6)	0.93 (0.84, 1.03)	0.174		
Health facility	1415	999 (70.5)	1			
**Child birthweight (Kg)**						
< 2.5	113	72 (63.7)	1			
≥ 2.5	1498	1059 (70.7)	1.11 (0.96, 1.28)	0.154		
**BF support immediately after delivery by provider**						
No	611	438 (71.7)	1			
Yes	244	166 (68.0)	0.95 (0.86, 1.05)	0.303		
**Prelacteal feeding**						
No	1457	1088 (74.7)	1		1	
Yes	176	54 (30.7)	0.41 (0.33, 0.51)	< 0.001	0.42 (0.34, 0.53)	< 0.001
**Child given colostrum**						
No	68	40 (58.8)	1.20 (0.98, 1.46)	0.081		
Yes	1574	1108 (70.4)	1			

*EIBF* Early Initiation of breastfeeding, *BF* Breastfeeding, *HF* Health Facility, *CPR* Crude Prevalence ratio, *APR* Adjusted Prevalence Ratio

In multivariable analysis, the district of residence, mother’s education level, parity, and prelacteal feeding were the factors independently associated with early initiation of breastfeeding (Table 4). Prevalence of early initiation of breastfeeding was 16% (PR 0.84; 95% CI 0.76, 0.93) and 11% (PR 0.89; 95% CI 0.80, 0.98) lower for women in Same and Hai districts, respectively compared to those in Rombo district. Women with primary education level had a 9% higher prevalence of early initiation of breastfeeding compared to those who had no formal education (PR 1.09; 95% CI 1.003, 1.18). Women who had given birth to two children had a 12% higher prevalence of early initiation of breastfeeding compared to primiparous mothers (PR 1.12, 95% CI 1.02, 1.21). Mothers who gave their children prelacteal feeds had a 58% lower prevalence of early initiation of breastfeeding (PR 0.42; 95% CI 0.34, 0.53) compared to those who did not give.

## Discussion

The prevalence of early initiation of breastfeeding (EIBF) was 70%. District of residence, women education level, parity, and prelacteal feeding were the factors independently associated with early initiation of breastfeeding in this setting.

Based on the WHO classification of percentages of breastfeeding within 1 h after delivery as (0–29) poor, (30–49) fair, (50–89) good and (90–100) very good, the prevalence obtained in this study is good [25] and higher than that reported in a study conducted in Rufiji, Kilombero and Ulanga districts if Tanzania [26] as well as national level findings of 51% [14]. However, these results show a missed opportunity to promote early initiation of breastfeeding because about 90% of deliveries in the Kilimanjaro region are assisted by the skilled birth attendants [14]. This call to healthcare providers to further assist and promote early initiation of breastfeeding. In low- and middle-income countries such as India, Bangladesh, Ethiopia, and South Sudan, the prevalence ranges between 40 and 51%, [6, 13, 26–29] which was lower than that reported in this study. The prevalence reported in Saudi Arabia was also low [21]. However, other studies in Malawi and South Sudan [11, 13] reported a much higher prevalence of early initiation of breastfeeding compared to the current study. These variations could be explained by differences in the study population, sample size, sampling procedures, study settings and period, sociodemographic characteristics such as access to health information issues, socio-economic status, infrastructures for enhancing health service accessibility, education level and cultural differences in breastfeeding practices [28, 30].

The prevalence of early initiation of breastfeeding was lower, particularly for women residing in the Same and Hai districts compared to women residing in the Rombo district. What causes such differences could not be fully established in this study. The variation found between districts in the same region could be explained by differences in environment, culture and beliefs. However, more should be done to assess differences in early initiation of breastfeeding prevalence between these districts.

In this study, women who had a primary education level had a higher prevalence of early initiation of breastfeeding compared to those with secondary education level or above. This was similar to the findings from Bangladesh, where the odds of early initiation of breastfeeding was lesser for women with secondary education level compared to those with non-formal education [31]. Fifty-seven percent of women in the current study with secondary education level were primiparous. In contrast, for women with primary education level, only 23% were primiparous, which could explain the low prevalence of early initiation of breastfeeding. Being educated can improve early initiation of breastfeeding practice due to increased awareness, however, in our context, educated mothers are wealthier than uneducated and can afford formula milk, so for them, it is a prestige to give their children prelacteal feeds.

Parity was observed to be positively associated with early initiation of breastfeeding. Women who have given birth to two children had a higher prevalence of early initiation of breastfeeding compared to primiparous. This is similar to findings from elsewhere [10–12, 22, 32, 33]. Women who have given birth more than once are likely to have more knowledge and experience on breastfeeding practices compared to nulliparous women, which is expected to influence their current practice [10]. Furthermore, delivery complications are more likely during the first pregnancy, which may end up in separation of the mother from the child, and can delay breastfeeding initiation [10].

Prelacteal feeding was negatively associated with early initiation of breastfeeding. This is consistent with findings from Nepal [17] and in East Ethiopia [34]. Studies have suggested that skilled and trained health workers can motivate mothers to practice early initiation of breastfeeding, explain the breastfeeding benefits, and can create awareness on the disadvantages of prelacteal feeding during routine postnatal care immediately after delivery [11, 34–37]. However, in this study, only 28.5% of 765 women who delivered in the health facilities received breastfeeding support immediately after birth, which could have contributed to prelacteal feeding, hence delay breastfeeding initiation. The health system should be strengthened to ensure that healthcare providers provide breastfeeding support to mothers immediately after delivery. There is a need to investigate the barriers at the health facilities that hinder healthcare providers from providing breastfeeding support to women soon after birth.

### Strength and limitations

By involving participants from most of the districts in the Kilimanjaro region, this study may provide the regional and sub-regional picture of early initiation of breastfeeding practice. However, our findings may not be generalized to other women across the country. This study might have been prone to recall and social desirability bias due to the self-reporting of time of breastfeeding initiation by women. This could have led to underestimation or overestimation of early initiation of breastfeeding practice in the region.

## Conclusions

The prevalence of early initiation of breastfeeding in the Kilimanjaro region was 70%. Though this estimate is good compared to WHO recommendations, it is still below the national targets. The district of residence, maternal education level, parity, and prelacteal feeding were the factors associated with EIBF in this study. To improve early initiation of breastfeeding, healthcare providers at reproductive and child health clinics should emphasize the impact of prelacteal feeding during and breastfeeding support immediately after delivery, particularly women who have given birth for the first time and those with secondary education and above. Interventions to improve early initiation of breastfeeding needs to be strengthened especially in Same and Hai districts. Furthermore, there is a need for qualitative study Same and Hai districts to understand reasons that delays initiation of breastfeeding which eventually leads to low prevalence of early initiation of breastfeeding.

## Data Availability

Please contact the author for data requests.

## Abbreviations

ANC: Antenatal Care
EIBF: Early initiation of breastfeeding
KCMUCo: Kilimanjaro Christian Medical University College
WHO: World Health Organization
